# Structures and determinants of soil microbiomes along a steep elevation gradient in Southwest China

**DOI:** 10.3389/fmicb.2024.1504134

**Published:** 2025-01-06

**Authors:** Ting Li, Ziyan Gao, Ping Zhou, Mingmin Huang, Gangzheng Wang, Jianping Xu, Wangqiu Deng, Mu Wang

**Affiliations:** ^1^School of Ecology and Environment, Tibet University, Lhasa, China; ^2^State Key Laboratory of Applied Microbiology in Southern China, and Guangdong Provincial Key Laboratory of Microbial Culture Collection and Application, Institute of Microbiology, Guangdong Academy of Sciences, Guangzhou, China; ^3^Guangdong Nanling Forest Ecosystem National Observation and Research Station, Shaoguan, China; ^4^Xizang Agricultural and Animal Husbandry University, Nyingchi, China; ^5^Guangzhou Institute of Geography, Guangdong Academy of Sciences, Guangzhou, China; ^6^Department of Biology, McMaster University, Hamilton, ON, Canada

**Keywords:** subtropical forests, soil ecology, Qinghai-Tibet Plateau, altitudinal gradient, microbial communities

## Abstract

Soil microbial communities play a vital role in accelerating nutrient cycling and stabilizing ecosystem functions in forests. However, the diversity of soil microbiome and the mechanisms driving their distribution patterns along elevational gradients in montane areas remain largely unknown. In this study, we investigated the soil microbial diversity along an elevational gradient from 650 m to 3,800 m above sea level in southeast Tibet, China, through DNA metabarcode sequencing of both the bacterial and fungal communities. Our results showed that the dominant bacterial phyla across elevations were Proteobacteria, Acidobacteriota and Actinobacteriota, and the dominant fungal phyla were Ascomycota and Basidiomycota. The Simpson indices of both soil bacteria and fungi demonstrated a hollow trend along the elevational gradient, with an abrupt decrease in bacterial and fungal diversity at 2,600 m a.s.l. in coniferous and broad-leaved mixed forests (CBM). Soil bacterial chemoheterotrophy was the dominant lifestyle and was predicted to decrease with increasing elevation. In terms of fungal lifestyles, saprophytic and symbiotic fungi were the dominant functional communities but their relative abundance was negatively correlated with increasing elevation. Environmental factors including vegetation type (VEG), altitude (ALT), soil pH, total phosphorus (TP), nitrate nitrogen (NO_3_^−^-N), and polyphenol oxidase (ppo) all exhibited significant influence on the bacterial community structure, whereas VEG, ALT, and the carbon to nitrogen ratio (C/N) were significantly associated with the fungal community structure. The VPA results indicated that edaphic factors explained 37% of the bacterial community variations, while C/N, ALT, and VEG explained 49% of the total fungal community variations. Our study contributes significantly to our understanding of forest ecosystems in mountainous regions with large elevation changes, highlighting the crucial role of soil environmental factors in shaping soil microbial communities and their variations in specific forest ecosystems.

## 1 Introduction

As an important component of ecosystems, soil microorganisms profoundly affect ecological functions, particularly in nutrient cycling, ecological succession, and the maintenance of biodiversity (Banerjee and van der Heijden, 2023). Soil microbial communities often show temporal and spatial patterns that are impacted by their biotic and abiotic factors such as geography and climate. Indeed, over short spatial distances and altitudinal gradients, climate, vegetation and soil properties can vary greatly (Saleem et al., 2019). Such changes can impact the soil physical and chemical properties which in turn can potentially impact the soil microbial communities and their associated plant and animal communities. However, our understanding of how large elevation change over a short spatial distance impact soil microbial communities remains very limited.

Previous studies have shown that soil pH, altitude, carbon-to-nitrogen ratio (C/N), vegetation type (VEG), soil temperature and available phosphorus as major drivers of the distributional patterns of soil microorganisms (Banerjee and van der Heijden, 2023). Among the major microbial groups in soil, bacterial diversity is often shaped by soil pH, temperature, soil organic matters, and moisture, while fungal diversity is mainly related to plant diversity, productivity, soil moisture (Wu et al., 2024).

Globally, the latitudinal diversity gradient with more plant and animal species toward the equator than away from the equator is a dominant pattern of their distributions (Economo et al., 2018). However, the soil bacterial communities seem to follow a dissimilar pattern. Specifically, bacterial diversity increases from the South Pole to the equator but shows no significant difference across the Northern Hemisphere (Delgado-Baquerizo et al., 2016). Numerous studies have revealed that bacterial diversity is higher in mid-latitude regions but decreases both at the poles and the equator (Bahram et al., 2018). For fungi, the diversity patterns are similar between hemispheres, with a strong decrease as latitude increases (Tedersoo et al., 2014). In contrast, other evidence indicates that fungal diversity is higher at higher latitudes (Větrovský et al., 2019). Ectomycorrhizal fungi, as one of the important fungal components, form various mutualistic symbioses with many tree species, and their geographical distribution patterns are similar to those of bacteria, with higher diversity in middle and upper latitudes but lower at the poles and the equator (Tedersoo et al., 2014). In summary, the distribution of soil microbial communities follows latitude-dependent patterns.

Analogous to latitudinal temperature patterns, elevation-dependent temperature differences are often regarded as the main cause for the observed differences in vegetation along elevational gradients. The vegetation differences can significantly influence the local climate and indirectly affect soil properties, leading to changes in the diversity of soil microorganisms (Pepin et al., 2015). However, patterns of elevation-dependent microbial diversity are often complex (Li et al., 2022). With increasing elevation, the diversity of soil microbial communities may increase, decrease, or a mixture of increase and decrease depending on specific sites (Yang et al., 2022). For example, soil microbial alpha-diversity exhibited a hollow pattern across elevations (2,980–4,050 m a.s.l.) in the Southern Himalayas of China (Yang et al., 2022). In contrast, soil bacterial and fungal community diversities monotonically increased across elevations (2,200–3,300 m a.s.l.) in the northwest Yunnan plateau, China, or showed a monotonically decreasing pattern along a higher elevational gradient (4,328–5,228 m a.s.l.) in the Southwestern Tibetan Plateau, China, or a hump-backed pattern with increasing elevation (1,000–3,760 m a.s.l.) on Mount Fuji, Japan (Singh et al., 2012; Shen et al., 2019; Cai et al., 2020). Non-consistent conclusions regarding changes in soil microbial communities across elevations have been reported (Dai et al., 2021). Specific local ecological features were often found to play a more important role than elevation change alone.

Medog County is under the jurisdiction of Nyingchi Prefecture in southeast Tibet. Geographically, it lies in the lower reaches of the Yarlung Tsangpo River on the southern slope of the Himalaya–Kangri Karpo Mountains and adjoins India. As the lowest elevation area in the Tibetan Plateau and a tropical monsoon rainforest zone, Medog County is characterized by a subtropical humid climate, with limited human disturbance in local forests (Ren et al., 2024). From the lowest elevation of Medog County to the Galongla Mountains, part of the Himalaya–Kangri Karpo Mountains, their direct horizontal distance is a relatively short (180 km) but with a significant elevational span (200–4,800 m a.s.l.) (Hu et al., 2020). While most previous studies covered elevation gains of about 1,000 m to less than 3,000 m, the Medog to Galongla range stretches 4,600 m elevation change and provides an excellent experimental platform for studying the diversity and community of soil microorganisms spanning a large elevation gradient. In addition, at present, there is limited information on soil bacterial and fungal communities and their driving factors in the montane forests in southeast Tibet. The aims of our study are to (i) reveal the patterns of soil bacterial and fungal diversity and community structures across elevations in Medog County, and (ii) identify the primary driving factors influencing soil microbial communities. Our results contribute to identifying potential mechanisms for maintaining montane ecosystems in relation to climate change.

## 2 Materials and methods

### 2.1 Site description and soil sampling

**Figure 1:**
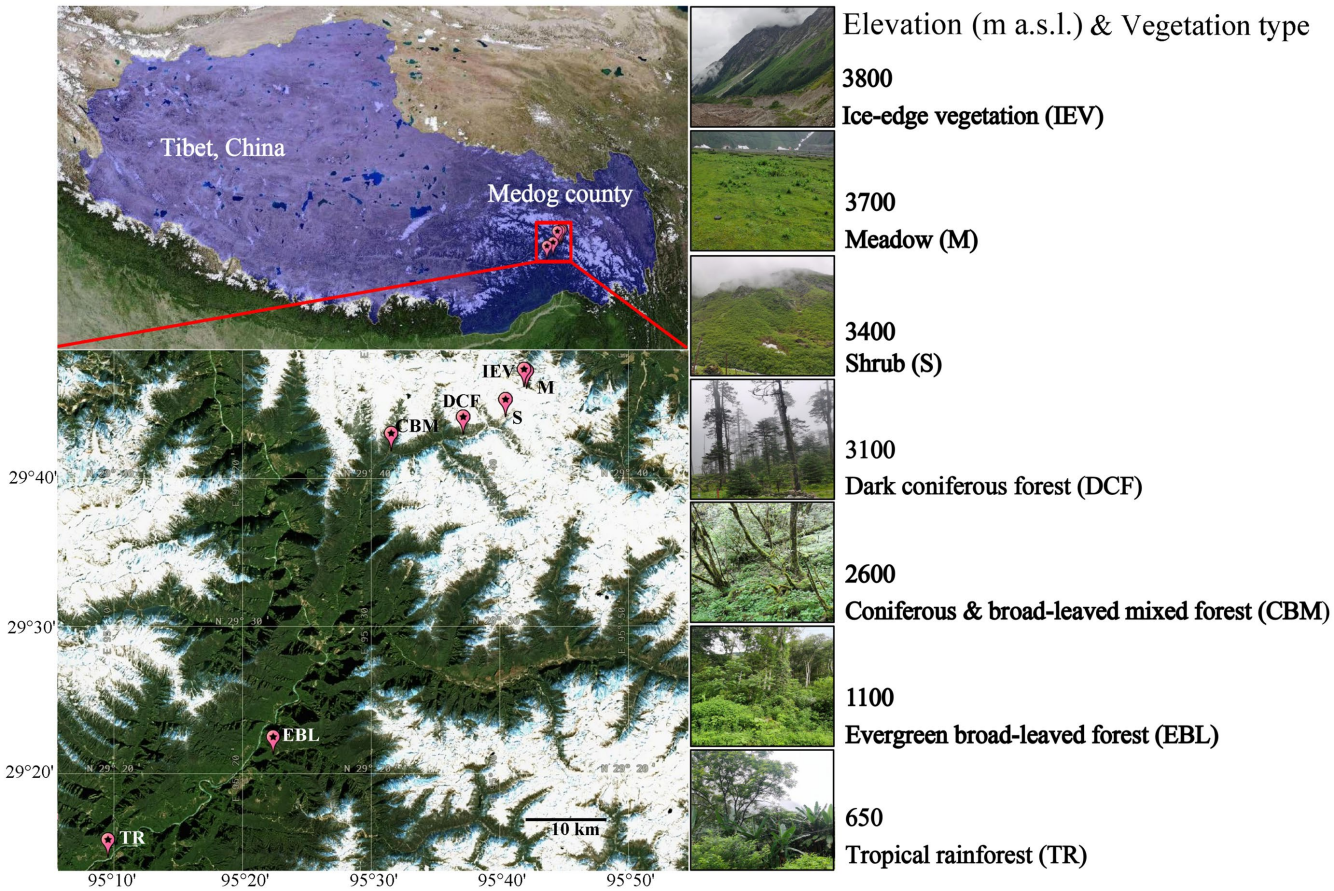
Geographic location of sampling sites in the montane range of Medog county.

Due to accessibility difficulty at both the lowest and highest elevations across the study region, here we selected the lowest elevation at 650 m above sea level (a.s.l.) to highest elevation at 3,800 m a.s.l. (29°14′–29°46′ N, 95°9′–95°41′ E) from the Medog County, the lower reach of the Yarlung Tsangpo River in southeast Tibet, China, to the Galongla Mountains. Along the increasing elevation, there was a range vegetation types (Supplementary Tables S1). Specifically, with increasing elevation, seven vegetation types (VEG) are found: tropical rainforest (TR) at below 1,100 m, evergreen broad-leaved forest (EBL) (1,100–2,200 m), coniferous and broad-leaved mixed forest (CBM) (2,200–3,000 m), dark coniferous forest (DCF) (3,000–3,400 m), shrub (S) (3,400–3,600 m), meadow (M) (3,600–3,750 m) and ice-edge vegetation (IEV) above 3,750 m (Figure 1).

In July 2023, soil samples were collected at seven sites across the seven vertical vegetation zones. We carefully selected sites with relatively consistent surroundings and limited anthropogenic disturbance on the southern slope of the Galongla Mountains for soil sample collections. Three standard plots, each measuring 3 m × 3 m, were established at each site. After removing the litter on the soil surface, a core of the soil at a depth of 0–10 cm was collected. Five cores from each plot were mixed homogeneously after removing gravel, fine roots, and other impurities using a 200-mesh sieve, packed into a sterile sealed bag, and then placed in a portable cryogenic refrigerator. A total of 21 soil samples were transported to the laboratory. Subsequently, each soil sample was divided into two subsamples: one for determining soil physical and chemical parameters, and the other for soil DNA extraction and DNA metabarcode high-throughput sequencing. The data on main vegetation and dominant plant species were referred from Yang and Zhou (2015) (Supplementary Tables S1). The NDVI (Normalized Difference Vegetation Index) (Sentinel-2 L2A)^1^ and soil information were documented (Supplementary Tables S2).

### 2.2 Soil physicochemical properties and enzyme activities measurement

We measured soil physicochemical variables, namely, soil pH, the soil water content (SWC), total carbon (TC), total nitrogen (TN), total phosphorus (TP), total potassium (TK), NH_4_^+^-N, NO_3_^−^-N and altitude (ALT) according to previous references (Cao et al., 2016; Naga Raju et al., 2017; Lu et al., 2023).

To determine the activity of soil enzymes, we measured urease (ure), sucrase (suc), acid phosphatase (pho), polyphenol oxidase (ppo), acid protease (pro), cellulase (cellu), amylase (amy) activities following the previously reported methods (Meng et al., 2020; Xie et al., 2021; Wang and Sheng, 2023).

### 2.3 Soil DNA extraction and high-throughput sequencing

Total DNA were extracted from 0.5 g of soil sample using the PowerSoil DNA Isolation Kit (MO BIO Laboratories Inc. Carlsbad, CA, United States) according to standard protocol (Cui et al., 2018). The concentrations of these DNA samples were quantified utilizing a NanoDrop2000C (Thermo Fisher Scientific, Waltham, MA, United States). DNA samples were checked by electrophoresis in 1% agarose gels and finally stored at −80°C for the subsequent high-throughput sequencing.

The V3-V4 region of 16S rDNA in bacteria were amplified by PCR using the primer pair of 338F (5′-ACTCCTACGGGAGGCAGCA-3′) and 806R (5′-GGACTACHVGGGTWTCTAAT-3′), and the ITS1 region (internal transcribed spacer 1) in fungi were amplified using primer pair of ITS1-F (5′-CTTGGTCATTTAGAGGAAGTAA-3′) and ITS1-R (5′-GCTGCGTTCTTCATCGATGC-3′). PCR amplification reaction system was: 2 × Taq PCR Mix 10 μL, each of forward and reverse primers 1 μL, DNA template 1 μL, add ddH_2_O to 25 μL. PCR amplification procedures were as follow: predenaturation at 95°C for 5 min, denaturation at 95°C for 30 s, annealing at 55°C for 30 s, extension at 72°C for 30 s, a total of 35 cycles, and extension at 72°C for 10 min (Zhang et al., 2017; Yang et al., 2019). Small fragment library preparations were processed on an Illumina HiSeq 2,500 sequencing platform which produced pair-end reads, by Beijing Biomark Biotechnology Co., Ltd., (Beijing, China).

### 2.4 Bioinformatic analysis

Raw data was processed for quality control, including removal of low-quality reads and length-based filtration, to generate high-quality sequences (Bolyen et al., 2019). First, use the Trimmomatic v0.33 software to filter the raw reads obtained from sequencing; then use the cutadapt 1.9.1 software for primer sequence recognition and removal, resulting in Clean Reads that do not contain primer sequences; second, employ the dada2 method within QIIME2 for denoising, where paired-end sequences are merged and chimeric sequences are removed, yielding the final valid data (Non-chimeric Reads) (Martin, 2011; Callahan et al., 2016; Bolyen et al., 2019). OTUs clustering (at a similarity level of 100%) were calibrated with mafft. Bacterial and fungal OTUs were annotated, against the database of Silva^2^ and UNITE (fungi),^3^ respectively. Initial sequences are available in the NCBI Genbank under accession numbers SRR31440083-31440103 (fungi) and SRR31440754-SRR31440774 (bacteria).

### 2.5 Statistical analyses

The top 30 soil microorganisms with relative high abundances at different taxonomic levels (phylum, class, order, family and genus) were calculated and showed by bar accumulation plots. Alpha and beta diversity indices were calculated for bacterial and fungal communities at different elevation. Simpson and Chao1 indices were used to measure the microbial diversity, and one-way ANOVA was used to assess their differences. Beta diversity of soil bacterial and fungal communities was analyzed utilizing non-parametric multidimensional scaling (NMDS) and tested by the Anosim. Ecologically relevant functions of bacterial taxa were predicted by FAPROTAX. Fungal OTUs were assigned to ecological guilds with the annotation tool FUNGuild. The statistical significance of inter-group difference was assessed using linear discriminant analysis effect size (LEfSe). The Redundancy Analysis (RDA) and Mantel-test verification were employed to analyze the significant relationship between soil microbial groups and environmental factors. Correlation heatmap along with corresponding *p*-values, was calculated to represent the strength and significance of correlations. The heatmap illustrated the clustering of species abundance in dominant bacterial and fungal communities at the different taxonomic unit levels (class, order, family, genus) along elevation gradient. To determine the proportion of community structure variation explained by specific environmental factors, variance partitioning analysis (VPA) was performed. The significant difference comparisons were conducted by the one-way ANOVA method. The differences between multiple comparisons were conducted by the Duncan method. The correlations between variables were analyzed by the Pearson correlation test. All statistical analyses were performed by SPSS software (Version 22.0, IBM, Chicago, United States). Canoco (Version 5.0, Microcomputer Power, Ithaca, NY, USA) and R statistical software, and GraphPad Prism 8.0, were employed to implement or visualize the correlations, cluster analysis.

## 3 Results

### 3.1 Soil bacterial and fungal communities

**Figure 2:**
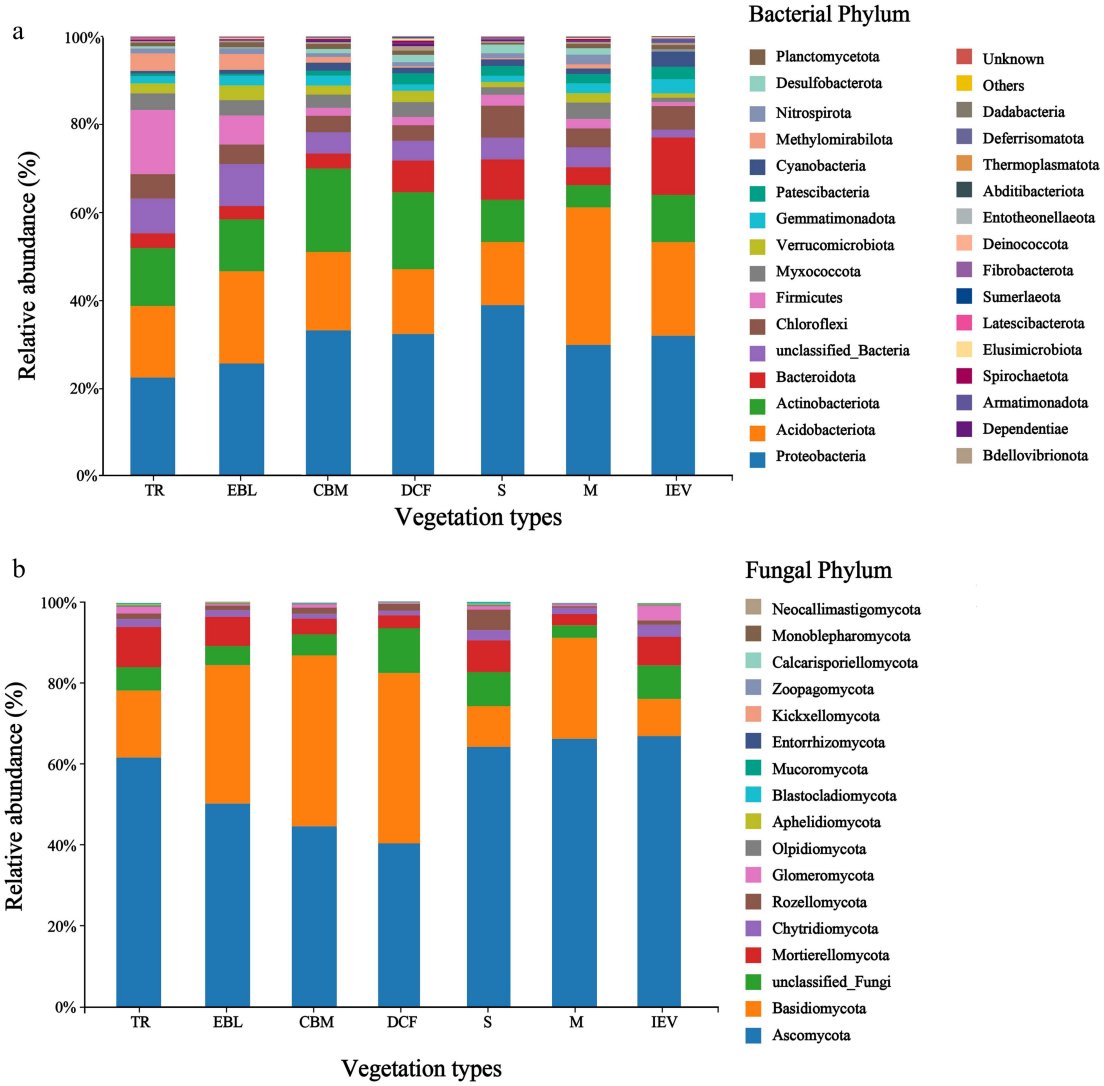
Soil bacterial **(A)** and fungal **(B)** communities at the phylum level at different elevations and vegetation zones in Medog county along an elevation gradient. From left to right on the X-axis with increasing elevation, TR, EBL, CBM, DCF, S, M, and IEV represent tropical rainforest, evergreen broad-leaved forest, coniferous and broad-leaved mixed forest, dark coniferous forest, shrub, meadow and ice-edge vegetation, respectively.

Our analyses identified a total of 35,719 and 8,970 OTUs for bacteria and fungi, respectively in our samples. The dominant soil bacterial community members at the phylum level were Proteobacteria (22.5–39.0%), Acidobacteriota (14.3–31.2%), and Actinobacteriota (5.1–19.0%), etc. Among all soil bacterial phyla, the relative abundance of the top three accounted for more than half of those at all elevations (52.0–70.0%). Proteobacteria had the highest relative abundance in shrubs (S) at 3,400 m, while more abundant Acidobacteriota were observed at 3,700 m with the meadow vegetation (M), and Actinobacteriota was the most abundant in coniferous and broad-leaved mixed forests (CBM) at 2,600 m (Figure 2A). Among the fungal phyla, Ascomycota and Basidiomycota dominated each community with a combined abundance of more than 74%, ranging from 74.2 to 91.4%, followed by Mortierellomycota. Ascomycota had a relatively higher proportion ranging from 40.1 to 67.1% than Basidiomycota, with site at 3,800 m a.s.l. with ice-edge vegetation (IEV) having the highest proportion of Ascomycota (67.1%). Basidiomycota was the second most abundant phylum after Ascomycota, ranging from 9.2 to 42.2% in the soil fungal communities, with the highest (42%) at 3,100 m a.s.l. in the dark coniferous forests (DCF). Basidiomycota was the least abundant at 9.2% at 3,800 m a.s.l. in the IEV zone (Figure 2B). The microbial community compositions at other taxonomic levels are shown in Supplementary Figure S1. As the altitude increases, the ratio of Ascomycota to Basidiomycota showed a hollow-shaped trend, with the lowest ratio in DCF (0.95) and the highest in IEV (7.31), indicating that Basidiomycota dominates in DCF, while Ascomycota dominates in IEV (Figure 2B).

### 3.2 Diversity indices of soil bacterial and fungal communities and their correlations with environmental factors

**Figure 3:**
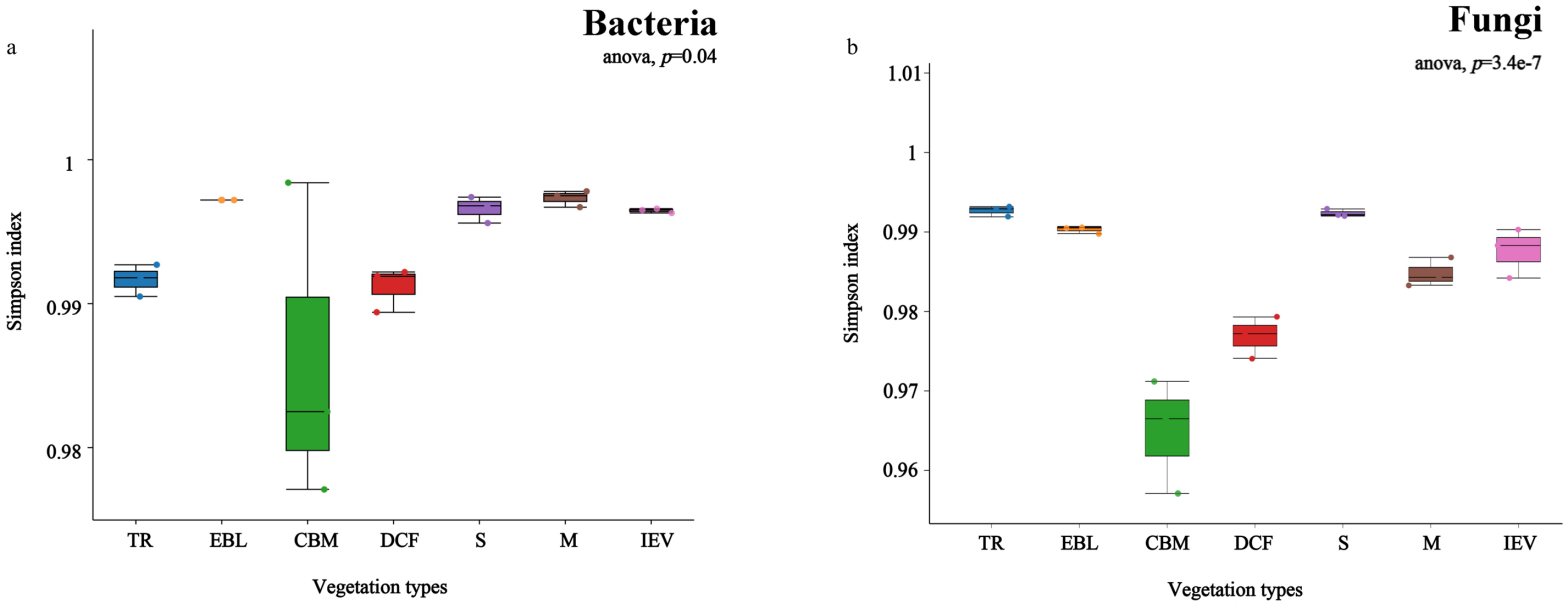
Alpha-diversity (Simpson index) of soil bacteria **(A)** and fungi **(B)** at different vegetation zones along an elevation gradient. From left to right on the X-axis with increasing elevation, TR, EBL, CBM, DCF, S, M and IEV represent tropical rainforest, evergreen broad-leaved forest, coniferous and broad-leaved mixed forest, dark coniferous forest, shrub, meadow and ice-edge vegetation, respectively.

The alpha diversity of soil bacteria in distinct vegetation zones indicated significant differences with Simpson index following a hollow pattern, reaching the lowest value at 2,600 m a.s.l. in CBM (Figure 3A). The Chao1 index of soil bacteria showed a declining trend with increasing elevation, attaining the highest richness at 650 m a.s.l. in tropical rainforests (TR) (Supplementary Figure S2). In case of soil fungi, the Simpson index also demonstrated a hollow pattern (Figure 3B). However, the Chao1 index of soil fungi displayed a double-hump fluctuation with elevation, showing maximum richness in DCF (Supplementary Figure S2).

**Figure 4:**
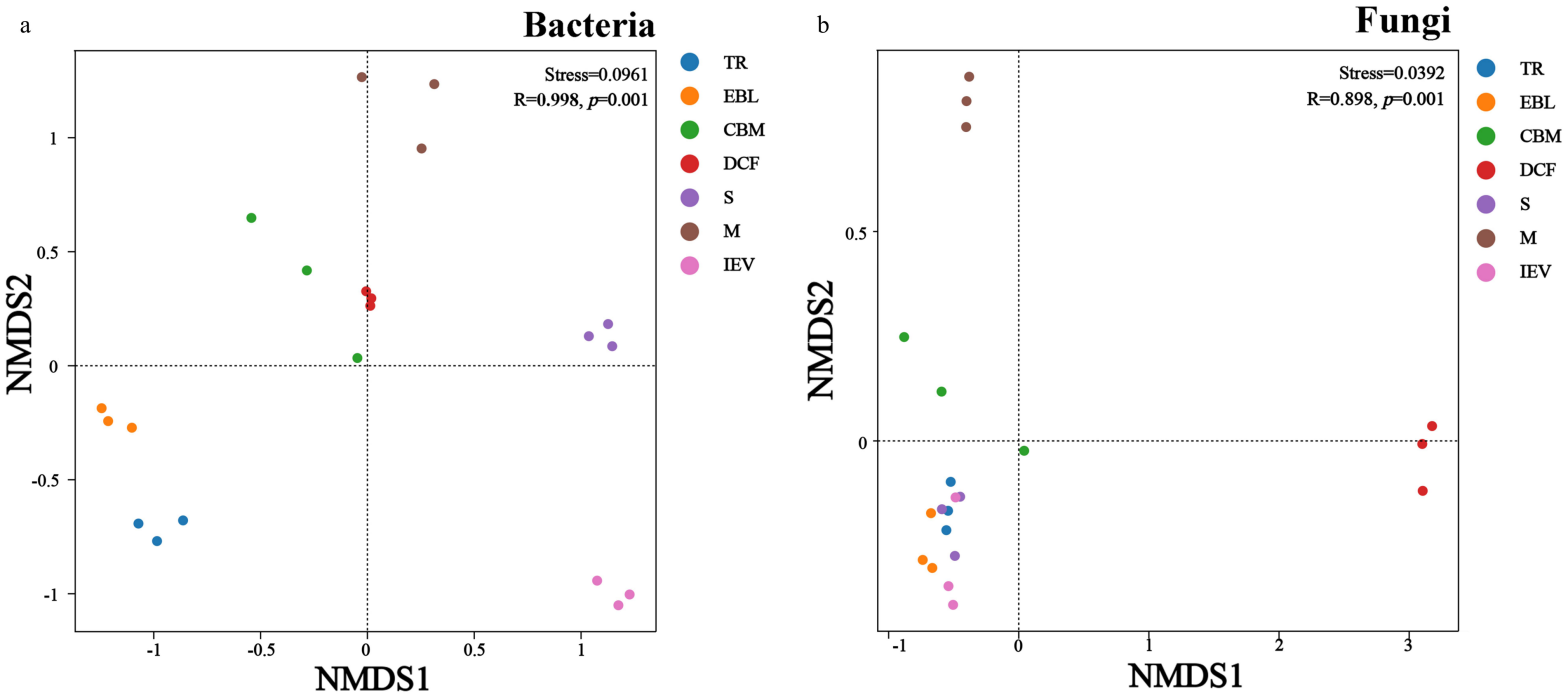
Beta-diversity (NMDS) of soil bacteria **(A)** and fungi **(B)**. TR, EBL, CBM, DCF, S, M, and IEV represent tropical rainforest, evergreen broad-leaved forest, coniferous and broad-leaved mixed forest, dark coniferous forest, shrub, meadow and ice-edge vegetation, respectively.

The beta diversity of soil bacteria showed that the community composition was clearly separated into three parts in the NMDS diagram (Figure 4A) as follows: first at the IEV site; second at the TR and EBL sites; and third at the remaining sites. The stress value of the NMDS was 0.0961, indicating that the goodness of fit allows for confident inferences. The physical distance between sites CBM and DCF was relatively short, whereas site IEV was isolated from other sites, likely contributing to their bacterial communities at CBM and DCF sites being more similar to each other, whereas the IEV site had more distinctive bacterial communities. Overall, the bacterial communities were more dispersed than the fungal communities. In terms of fungal communities, the NMDS ordination revealed that the DCF and M sites were significantly different from others, forming two separate clusters, whereas sites TR, EBL, S, and IEV were clustered together (Figure 4B). The stress value of the NMDS for the fungal diagram was 0.0392, indicating an excellent fitting degree. Using the ANOSIM test method, NMDS ordination showed that the R values in both bacterial and fungal communities were close to 1, and the *p*-values were both 0.001, consistent with elevation playing a significant role in microbial community structures in Medog county and the surround region.

**Figure 5:**
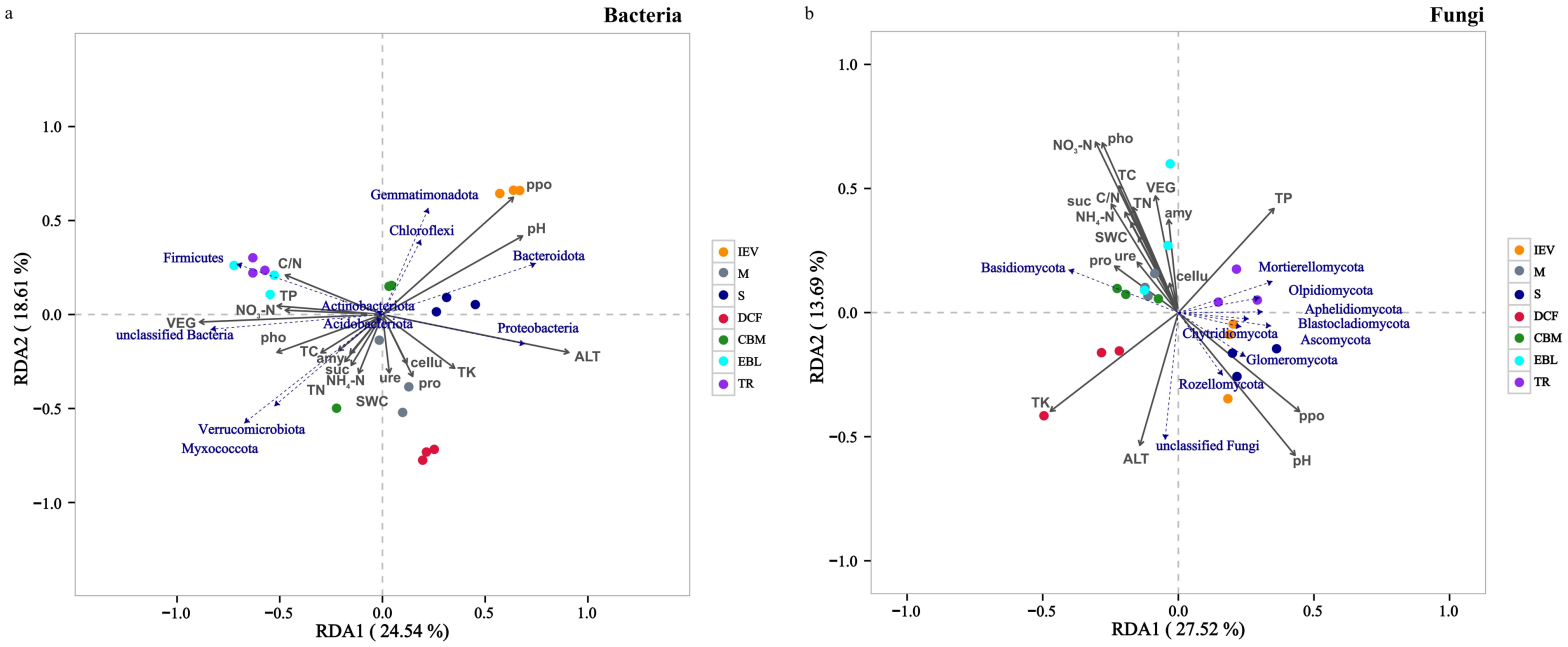
Redundancy analysis (RDA) demonstrating the effect of soil properties on community composition of soil bacteria **(A)** and fungi **(B)**. Abbreviations: TR, tropical rainforest; EBL, evergreen broad-leaved forest; CBM, coniferous and broad-leaved mixed forest; DCF, dark coniferous forest; S, shrub; M, meadow; IEV, ice-edge vegetation; pH, pH value; TC, total carbon; TN, total nitrogen; C/N, carbon nitrogen ratio; TP, total phosphorus; TK, total potassium; NH_4_^+^-N, ammonium nitrogen; NO_3_^−^-N, nitrate nitrogen; ure, urease; pho, polyphenol oxidase; suc, sucrase; ppo, acid phosphatase; cellu, cellulase; amy, amylase; pro, acid protease; SWC, soil water content; VEG, vegetation type.

**Figure 6:**
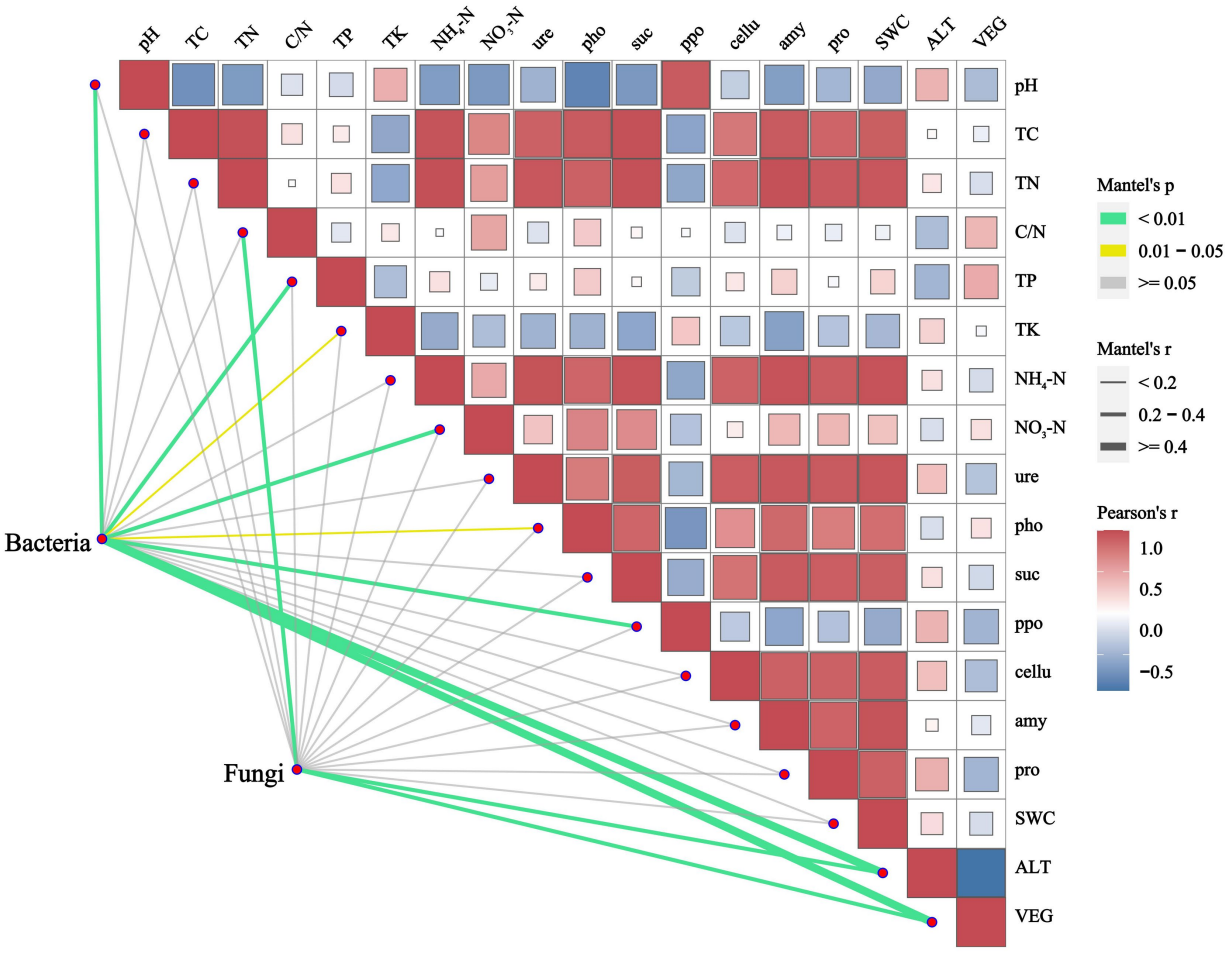
Correlation between environmental factors and microbial communities along an elevation gradient in southeast Tibet. Bacterial and fungal communities were related to total of 11 significantly differential environmental factors using Mantel-test analysis with r > 0.15, *p* < 0.05. Edge width corresponds to the Mantel’s r statistic for the corresponding distance correlations, and edge color denotes the statistical significance. Pairwise comparisons of environmental factors are shown, with a color gradient denoting Pearson’s correlation coefficient. pH, pH value; TC, total carbon; TN, total nitrogen; C/N, carbon nitrogen ratio; TP, total phosphorus; TK, total potassium; NH_4_^+^-N, ammonium nitrogen; NO_3_^−^-N, nitrate nitrogen; ure, urease; pho, polyphenol oxidase; suc, sucrase; ppo, acid phosphatase; cellu, cellulase; amy, amylase; pro, acid protease; SWC, soil water content; VEG, vegetation type.

RDA was conducted to analyze the relationships between soil microbial communities at the phylum level and environmental factors at different vegetation zones. The examined environmental factors could explain 43.15% of the variation in the dominant soil bacterial community, of which the first axis (RDA1) explained 24.54% and the second axis (RDA2) explained 18.61% (Figure 5A). Similarly, these factors could explain 41.21% of the variation in the dominant soil fungal community, with the first axis explaining 27.52% and the second axis explaining 13.69% (Figure 5B). Soil pH, ALT and ppo showed notably positive correlations with the bacterial community in the S and IEV zones, but negative correlations with those in the TR and EBL zones. However, TP, TK and pho were positively associated with those of TR and EBL zones. For bacterial community structures, Proteobacteria was strongly associated with ALT, whereas Bacteroidota, Chloroflexi and Gemmatimonadota were correlated with soil pH and ppo, and Firmicutes was associated with TP, C/N and NO_3_^−^-N. Interestingly, no statistically significant correlation was found between Acidobacteriota, Actinobacteriota and any environmental factors (Figure 5A). Regarding the fungal community, it was strongly correlated with ALT, C/N and VEG. Among them, ALT was positively correlated with those in the DCF, S, and IEV zones, but negatively correlated with those in the TR, EBL, and M zones. The C/N showed a significantly positive correlation with those of the EBL, CBM, and M zones, but a negative correlation with those of the S and IEV zones. At the fungal phylum level, Basidiomycota showed a distinct pattern, with soil pH, ppo, TP, and ALT displaying negative correlations, while Ascomycota, Mortierellomycota, Chytridiomycota and Rozellomycota exhibited positive correlations with soil pH, ppo, and TP (Figure 5B). Based on the Mantel-test verification, environmental factors VEG, ALT, pH, TP, NO_3_^−^-N and ppo showed strongly significant correlations with the bacterial community, whereas VEG, ALT, and C/N were highly significantly correlated with the fungal community (Figure 6). In addition, the results of VPA indicated that edaphic factors, including soil pH, TP, NO_3_^−^-N, ppo, TK, and pho, explained a total of 37% of the bacterial community variation, followed by ALT (7%) and VEG (6%), individually. As strongly significant correlation factors, C/N, ALT, and VEG explained a total of 49% of the fungal community variation, each individually explaining 6, 36, and 45%, respectively. However, two out of three factors were pairwise independent (Supplementary Figure S3).

### 3.3 Intergroup comparisons reveal major biomarker taxa distinguishing vegetation zones

The LEfSe analysis identified biomarker taxa contributing to statistically significant differences in bacterial and fungal communities between vegetation zones. Here, branch maps were used to identify discriminative taxa in soil bacteria and fungi among vegetation types. An LDA score of 4 was set to identify bacterial and fungal biomarkers at taxonomic levels from phylum to genus. For bacteria, the LDA scores of 71 taxa were greater than 4, among which the IEV zone possessed the maximum number of bacterial biomarkers (24 taxa), followed by the M zone (15 taxa) and TR zone (12 taxa), while no discriminative bacterial biomarker taxon was observed in the DCF zone. In terms of bacterial genera, Bacillus, an unclassified genus under Xanthobacteraceae, Acinetobacter, an unclassified genus under Acidobacteriales and Sphingomonas were significant biomarker taxa in the TR, CBM, S, M, and IEV zones, respectively (Supplementary Figure S4). With regard to fungi, 80 taxa were identified as biomarker taxa under the same analytical parameters. The statistically significant fungal biomarkers in the DCF zone had the greatest number of biomarker taxa (21 taxa), followed by the S zone (16 taxa) and the IEV zone (13 taxa). In terms of fungal genera, *Mortierella*, *Hebeloma*, *Russula*, *Inocybe*, *Aspergillus*, *Archaeorhizomyces* and *Diversispora* were the significant biomarkers in the TR, EBL, CBM, DCF, S, M, and IEV zones, respectively (Supplementary Figure S4).

### 3.4 Function prediction of soil bacterial and fungal communities

**Figure 7:**
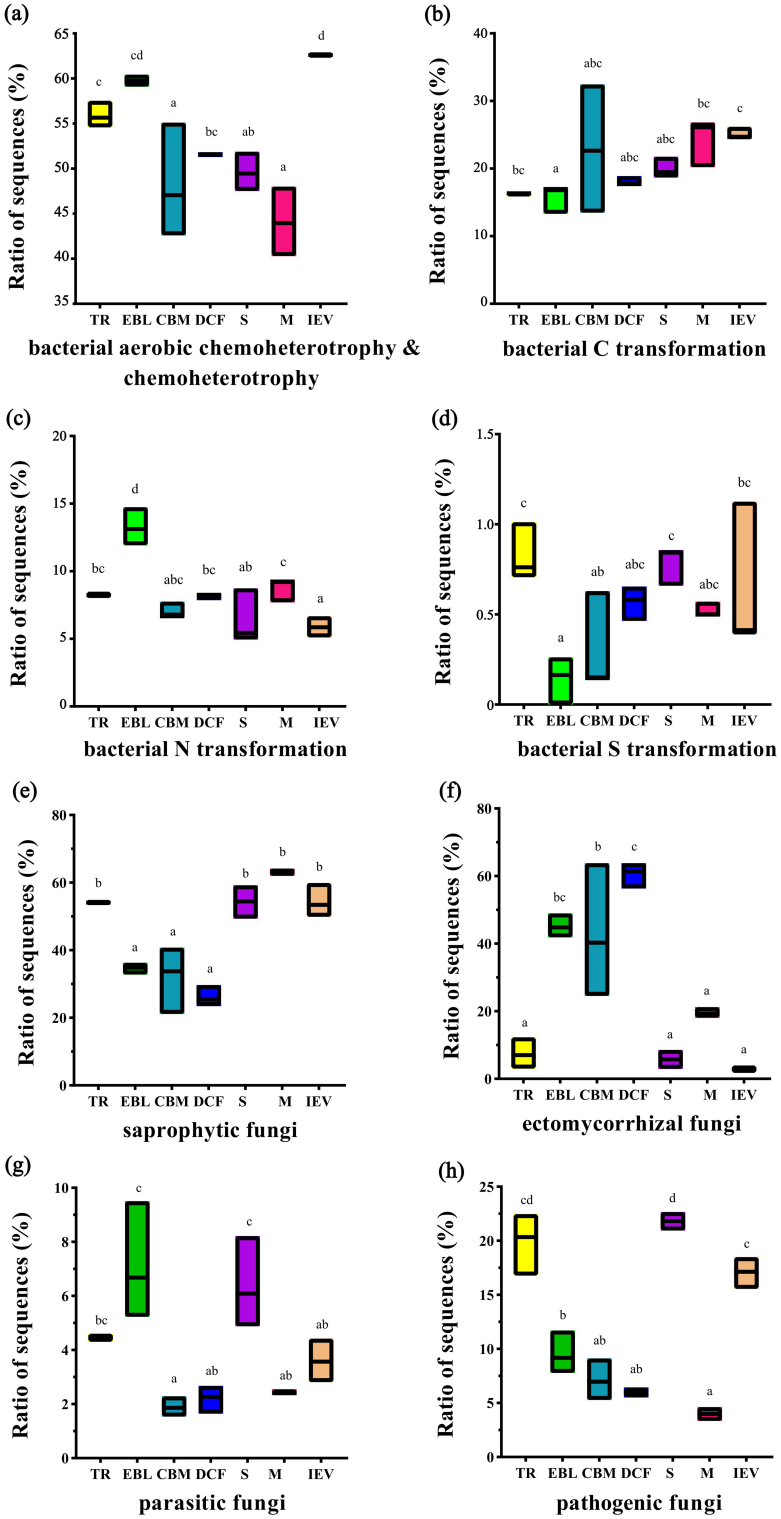
Functional prediction of bacteria **(A–D)** and fungi **(E–H)** at different vegetation zones along an elevation gradient. From left to right on the X-axis with increasing elevation: TR, EBL, CBM, DCF, S, M and IEV represent tropical rainforest, evergreen broad-leaved forest, coniferous and broad-leaved mixed forest, dark coniferous forest, shrub, meadow and ice-edge vegetation, respectively. Lowercase letters indicate statistically significant differences at *p* < 0.05.

FAPROTAX analysis predicted 61 metabolic functional assemblages involving bacterial transformation of C (including chemoheterotrophy and aerobic chemoheterotrophy), N, and S. Among them, soil bacterial chemoheterotrophic functions were dominant, with an average sequence mapping ratio of 52.86% across all sites. Overall, the mapping ratio of sequences related to bacterial chemoheterotrophic functions showed a roughly decreasing trend with increasing elevation, while the trends for C and S transformations were opposite. Bacterial N transformation had a similar mapping ratio of sequences (approximately 7.4%) across all sites, except for the EBL zone, which was 13.3% (Figures 7A–D). As for fungi, the FUNGuild was used for functional prediction, including saprophytic, symbiotic (ectomycorrhizal), parasitic, and pathogenic fungi. Saprophytic and symbiotic (ectomycorrhizal) fungi, as dominant functional communities, accounted for an average of 45.65 and 30.41% of the total sequences, respectively, across all sites. The mapping ratio of sequences in saprophytic fungi presented a hollow pattern with increasing elevation, whereas ectomycorrhizal fungi showed a hump-backed trend, and parasitic fungi exhibited a double-hump fluctuation. Parasitic fungi, having the lowest ratio of sequences, accounted for an average proportion of 3.91% across all sites. The mapping ratio of pathogenic fungal sequences showed an irregular fluctuation with increasing elevation (Figures 7E–H).

### 3.5 Co-occurrence network analysis of dominant soil bacterial and fungal communities

The OTU linking to soil bacteria phylum formed a complex biological network among vegetation zones. The biological network diagram indicated the complex relationship. There were 87 nodes in the network, with 362 edges including 335 (92.5%) positive correlation and 27 (7.5%) negative correlation. The average path length between all nodes was 3.01, the longest distance was 6 edges, the modularity index was 0.55, and the clustering coefficient was 0.71. Likewise, the OTU linking to the soil fungal community at phylum level composed an intricate network. There were 76 nodes, 270 edges in the fungal network with 99.25% positive correlation and 0.75% negative correlation. The average path length and longest distance were similar to those of bacteria. However, the fungal network had a lower modularity index (0.45), and a lower clustering coefficient index (0.61) (Supplementary Figure S5). These results implied that the dominant soil microorganisms at different sites of VEG and altitude show a distinct modular structure.

## 4 Discussion

### 4.1 Composition and diversity of the soil microbial community change with increasing elevation

The composition of the main bacterial communities in the soil is roughly the same across elevation gradients at the phylum level, with Acidobacteria, Proteobacteria, and Actinobacteriota as the most abundant phyla, regardless of land use or ecosystem types (Vélez-Martínez et al., 2023). In this region, Proteobacteria, Acidobacteria, and Actinobacteriota were the major phyla of soil bacteria, being almost identical with those from montane areas, such as the Greater Khinggan Mountains (Northeast China), the central Guizhou hilly areas, the Gongga Mountains (Southwest China) and the Tibetan plateau (Cui et al., 2019; Dai et al., 2023; Wang et al., 2023; Zhai et al., 2023). In this study, Proteobacteria was the most abundant bacterial phylum at all sites, particularly in shrubs, which had the lowest C/N ratio. Its trend was consistent with reports of copiotrophic strategies across an elevational gradient, with an inverse preference for abundances in ecosystems with high percentages of carbon (Cleveland et al., 2007). The soil with a high C/N ratio might be not favorable for the growth of bacteria (Siles and Margesin, 2016). Acidobacteria, one of the bacteria favored acidic conditions, was the second-most abundant soil bacterial phylum in all sites. Compared with other sites, the meadow sites shared more abundant Acidobacteria with the lowest soil pH (average 5.5) and the highest soil TC (average 101.3 g/kg). Although bacterial ecology has associated this phylum with oligotrophic strategies, recent findings suggest contrasting results (Kielak et al., 2016). Actinobacteriota showed a general trend of decreasing abundance across the elevational gradient. Especially in the alpine meadow ecosystem, Actinobacteriota showed the lowest abundance, corresponding to the lowest soil pH (Siles and Margesin, 2016). However, in this study, Actinobacteriota had no or inconspicuous correlation with any environmental factors, which was inconsistent with previous studies showing a positive correlation with pH (Siles and Margesin, 2016).

The most abundant fungal phyla in all sites were Ascomycota and Basidiomycota, which together accounted for more than 74% of the total sequences. Their abundances were similar to the soil fungal composition in Gongga Mountains, ranging from 1,600 to 3,900 m a.s.l. and the Yungas region, ranging from 400 to 3,000 m a.s.l. in the Andes Mountains in Argentina (Geml et al., 2014; Tian et al., 2017). However, these results were not in line with the soil fungal composition in Nanling Mountains and Changbai Mountains (both with the altitudes below 2,000 m a.s.l. and located in South China and North China respectively), which were dominated by Basidiomycota, followed by Ascomycota (Ping et al., 2016; Li et al., 2023b). In this study, Ascomycota showed a reverse hump-shaped trend for abundance in response to increased elevation, and had the lowest abundance in DCF, whereas Basidiomycota displayed a hump trend with the increasing elevation. Notably, the class Agaricomycetes was more abundant in DCF at 3,100 m a.s.l. and less abundant at the sites with lower or higher elevations. Our results were not congruent with the findings reported by Vélez-Martínez et al., which found that soil bacteria and fungi communities from tropical regions in South America showed an ascending trend and a declining trend, respectively, following the increase in elevation (Vélez-Martínez et al., 2023) These biogeographic variations in bacteria and fungi with elevation are mainly attributable to climatic factors in different ecoregions (Větrovský et al., 2019).

In this study, the soil bacterial *α*-diversity, as measured by the Simpson index, exhibited a hollow pattern, with the lowest value (turning point) at 2,600 m a.s.l. in the CBM zone. This pattern was consistent with those reported in the Gongga Mountains, where soil bacterial diversity showed a stair-step pattern. Specifically, there was an abrupt decrease in bacterial diversity between 2,600 and 2,800 m, while non-significant changes occurred at either lower (1,800–2,600 m) or higher (2,800–4,100 m) elevations (Li et al., 2018). However, the α-diversity of fungi in Gongga Mountains showed a slow downward pattern with increasing elevation, which was inconsistent with the findings of this study that fungi exhibited a hollow pattern. Our findings could provide another fundamental contribution to the understanding of fungal diversity patterns in mountain ecosystems.

### 4.2 Predictive functional annotation of the microbiome

The C and N cycles in the terrestrial ecosystem and their regulation mechanisms are hot topics in soil ecology and global change ecology (Meng et al., 2020). The functional annotation of bacteria revealed that the functions with the highest number of sequences were aerobic chemoheterotrophy and chemoheterotrophy. Both were more abundant at lower elevational sites, especially in EBL (1,100 m a.s.l.) and TR (650 m a.s.l.) sites and decreased as elevation increased. This observation was consistent with a recent study from the Colombian forest, which was mainly attributed to differences in vegetation among natural systems (Vélez-Martínez et al., 2023). Abundant chemoheterotrophy in soil indicates that many bacteria cannot fix carbon and have to obtain carbon and energy by oxidizing preformed organic compounds (Zhang et al., 2018). We also noticed that the relative abundance of chemoheterotrophs corresponded roughly with the concentration of total C in soil along elevation gradient. For the annotated function of N transformation, the abundance of sequences was generally similar among all sites, which was consistent with the global biogeography of microbial nitrogen-cycling traits in soil (Nelson et al., 2016). This result also indirectly supported the notion that the soil bacteria can use various N functional pathways to participate in the N cycle; however, they had low-abundance sequences compared to those from the C cycle (Nelson et al., 2016). Sulfur (S) is a macroelement required for life, and S deficiency limits plant growth. Microorganisms carry out several essential steps in the recycling of organic and inorganic S in soils (Santana et al., 2021). In this study, the abundance of sequences mapping to S transformation generally increased with increasing elevation, which is in line with previous research results (Stanko and Fitzgerald, 1990).

In our study, saprotrophic fungal diversity showed a hollow pattern with increasing elevation in soils. This is not consistent with studies in Changbai Mountain, Northeast China, where diversity increased steadily with increasing elevation (Yang et al., 2021). However, both sites had a higher ratio of saprotrophic fungal sequences at high elevation. The higher diversity of herbaceous plants at high elevation habitats possibly contributed to the higher diversity of soil saprotrophic fungi by providing more diverse habitats and more plant chemical inputs (Waring et al., 2015). Saprotrophic fungi are highly correlated with carbon content (Marañón-Jiménez et al., 2021). In meadow ecological regions, there is a higher carbon content, which correlates with a higher abundance of saprotrophic fungi, aligning with the findings of our study (Li J. S. et al., 2023). Ectomycorrhizal fungi showed a hump-shaped trend with increasing elevation, declining in higher elevation sites, which was in line with those in forests of Changbai Mountain, Hyrcanian forests of northern Iran, and the Front Range of the Canadian Rockies, where climate factors were proposed as the primary mechanism to explain the variations (Kernaghan and Harper, 2001; Bahram et al., 2012; Bai et al., 2020). Furthermore, abundant ectomycorrhizal fungi including *Inocybe*, *Russula*, *Laccaria,* and *Thelephora* widely existed in intermediate altitude regions, such as the EBL, CBM, and DCF sites (Supplementary Figure S1). It is also verified that the diversity of ectomycorrhizal fungi is strongly related to the mixed-forest in the middle-gradient sites (Martin et al., 2016; Bai et al., 2020). Ectomycorrhizal fungi provide a protective shield for plant roots against pathogenic microorganisms, soil pollutants, and even nuclear radiation; this feature enhances the plants’ environmental resilience. Moreover, these fungi facilitate the direct uptake of nitrogen and phosphorus from soil organic matter, which not only improves plant survival by increasing nutrient acquisition efficiency but also makes plants more adaptable to harsh conditions such as drought, low nutrient availability, and cold. Ectomycorrhizal fungi also protect biodiversity in high-latitude temperate regions through their interactions with plants (Tedersoo et al., 2020; Cheng et al., 2023; Wu et al., 2023; Guo et al., 2024; Song, 2024). The relative abundance of soil pathogenic fungi and ectomycorrhizal fungi displayed opposite elevation patterns (Figure 7). This is consistent with previous findings that pathogenic fungi and ectomycorrhizal fungi in soil prefer different ecological niches. Soil pathogenic fungi may rapidly proliferate in warmer conditions, such as in tropical forests, leading to increased plant diseases (Looby and Treseder, 2018). Similarly, the relative abundance of pathogenic fungi in soil with shrubs was significantly higher than in other VEG, implying that potential plant pathogens could cause root rot and other diseases in shrubs. However, the abundance of soil pathogenic fungi in shrubs was not related to elevation, a pattern different from previous study in dry valley shrubs at lower elevations (1,663–1,848 m a.s.l.) in the eastern Qinghai–Tibetan Plateau (Chen et al., 2022).

### 4.3 Associations of microorganisms with physicochemical parameters

Environmental conditions have been reported as major factors affecting elevation patterns of microbial communities (Donhauser and Frey, 2018). In this study, pH, TP, NO_3_^−^-N, ALT, and VEG were identified as important factors influencing bacterial communities along elevation gradient from 600 m to 3,800 m a.s.l. Previous studies have emphasized the roles of soil properties (such as pH, soil TC, TP, etc.) on microbial community composition (Siles and Margesin, 2016). Factors such as pH and NO_3_^−^-N, can contribute to N deposition on soil microbial communities. Thus, soil bacterial communities are generally more sensitive than fungal communities to short-term nitrogen deposition. Likewise, bacterial communities are more vulnerable to environmental changes than fungal communities (Siles and Margesin, 2016). Soil bacterial abundance was positively correlated with soil pH and TP and negatively correlated with soil TC (Yan et al., 2021). Previous findings also indicated altitudes and VEG have the greatest effect on the bacterial communities in subtropical forests (Cui et al., 2022; Jia et al., 2024). Another report based on the altitude changes in the Gongga Mountain area revealed that climate change could not alter the bacterial communities if the changes were not significant enough to alter vegetation and soil properties (Sun et al., 2020). In other words, VEG is a pivotal driving factor affecting bacterial communities.

In terms of fungi, soil C/N was the main driving factor affecting the fungal communities. This finding is consistent with previous research showing that the structure of the fungal communities significantly differed along altitude, owing to the changes in soil C/N (Siles and Margesin, 2016). However, soil pH was of minor significance for fungal community composition. Similar conclusions were drawn, indicating that the effect of pH on fungal community composition was lower than on bacterial communities (Rousk et al., 2010; Wang et al., 2015). Changes in VEG with altitude lead to significant changes in soil fungal community composition, as this is tightly coupled with vegetation properties through host specificity and the production of different organic substrates (Peay et al., 2013). The effect of altitude gradients on the dominant fungal taxa may be important for interpreting the altitude patterns of fungal diversity and understanding ecosystem function. For example, Ascomycota and Basidiomycota showed opposite distribution patterns along altitude (Meng et al., 2013; Yao et al., 2017). Ascomycota is stress-tolerant, meaning they are more resilient in harsh habitats (dry or cold) than other fungal taxa. Meanwhile, Basidiomycota may have a stronger competitive advantage than other fungal taxa in better environmental conditions (Li T. et al., 2023). These conclusions can explain the highest proportion of Ascomycota found in IEV sites, and the more abundant Basidiomycota in CBM and DCF sites.

## 5 Conclusion

Unraveling the composition and structure of soil microbial communities along an elevational gradient in montane ecosystems of southeast Tibet, China, and exploring the mechanisms responsible for microbial community assembly are critical for understanding global soil microbial distribution patterns and how they might respond to climate changes. We found that the dominant soil bacteria were Proteobacteria, Acidobacteriota and Actinobacteriota, while the dominant soil fungi were Ascomycota and Basidiomycota. In this area, both soil bacteria and fungi displayed a hollow trend along the elevational gradient, with an abrupt decrease at 2,600 m a.s.l in coniferous and broad-leaved mixed forests. Environmental factors significantly influenced the variations of soil microorganism communities. For bacteria, this influence was mainly driven by vegetation type (VEG), altitude, soil pH, total phosphorus, nitrate nitrogen and polyphenol oxidase. However, for fungi, VEG, altitude and carbon nitrogen ratio were the mainly driving factors. Our study contributes to a deeper understanding of how environmental factors shape soil microbial diversity and ecosystem functioning in a specific montane area. Future efforts in Medog area could focus on analyzing the metagenome within these regions to identify the metabolic pathways and gene clusters that are unique at each vegetation type, including identifying their potential novel properties such as antimicrobials to forestry and human health.

## Data Availability

The raw data associated with this article is publicly available and their accession numbers are shown in section 2.4.

## Footnotes

^1^ https://browser.dataspace.copernicus.eu/

^2^ https://www.arb-silva.de/bacteria

^3^ https://unite.ut.ee

## Supplementary material

Supplementary Figure S1 Soil bacterial and fungal communities in genus, family, order and class level at different typical vegetations in Medog montane zones. TR, EBL, CBM, DCF, S, M, and IEV represent tropical rainforest, evergreen broad-leaved forest, coniferous and broad-leaved mixed forest, dark coniferous forest, shrub, meadow and ice-edge vegetation, respectively

Supplementary Figure S2 Alpha-diversity (Chao1 index) of soil bacteria and fungi along the elevation. TR, EBL, CBM, DCF, S, M, and IEV represent tropical rainforest, evergreen broad-leaved forest, coniferous and broad-leaved mixed forest, dark coniferous forest, shrub, meadow and ice-edge vegetation, respectively

Supplementary Figure S3 Variance partitioning analysis (VPA) analysis of the contribution of environmental factors to bacterial communities and the contribution of environmental factors to the true community

Supplementary Figure S4 Linear discriminant analysis effect size (LEfSe) analyses of bacterial and fungal communities associated with the vegetation type zones. TR, EBL, CBM, DCF, S, M, and IEV represent tropical rainforest, evergreen broad-leaved forest, coniferous and broad-leaved mixed forest, dark coniferous forest, shrub, meadow and ice-edge vegetation, respectively; o, f and g represent order, family and genus, respectively

Supplementary Figure S5 The co-occurrence network analysis of dominant soil bacteria **(A)** and fungi **(B)** in phylum along the elevational gradient. Larger circles indicate higher relative abundance. Red lines represent positive correlation, while green lines represent negative correlation.
