# Supplementary figures and images for: Structures and determinants of soil microbiomes along a steep elevation gradient in Southwest China
Source: Front Microbiol. 2025 Jan 6;15:1504134. doi: 10.3389/fmicb.2024.1504134 (PMC11743684; doi:10.3389/fmicb.2024.1504134)

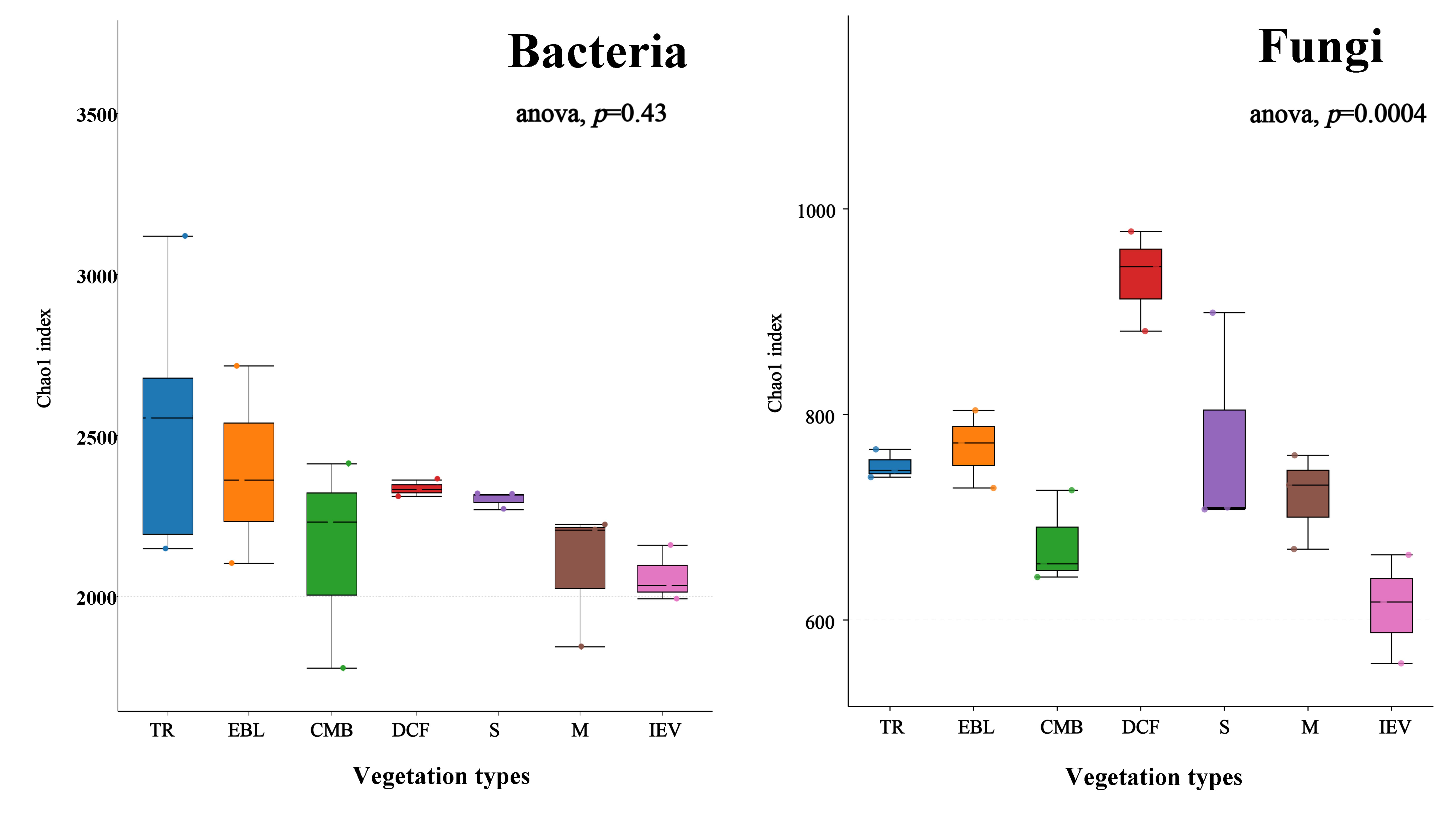

Supplement: Supplementary Figure S2 — Alpha-diversity (Chao1 index) of soil bacteria and fungi along the elevation. TR, EBL, CBM, DCF, S, M, and IEV represent tropical rainforest, evergreen broad-leaved forest, coniferous and broad-leaved mixed forest, dark coniferous forest, shrub, meadow and ice-edge vegetation, respectively [file Image_2.jpeg]

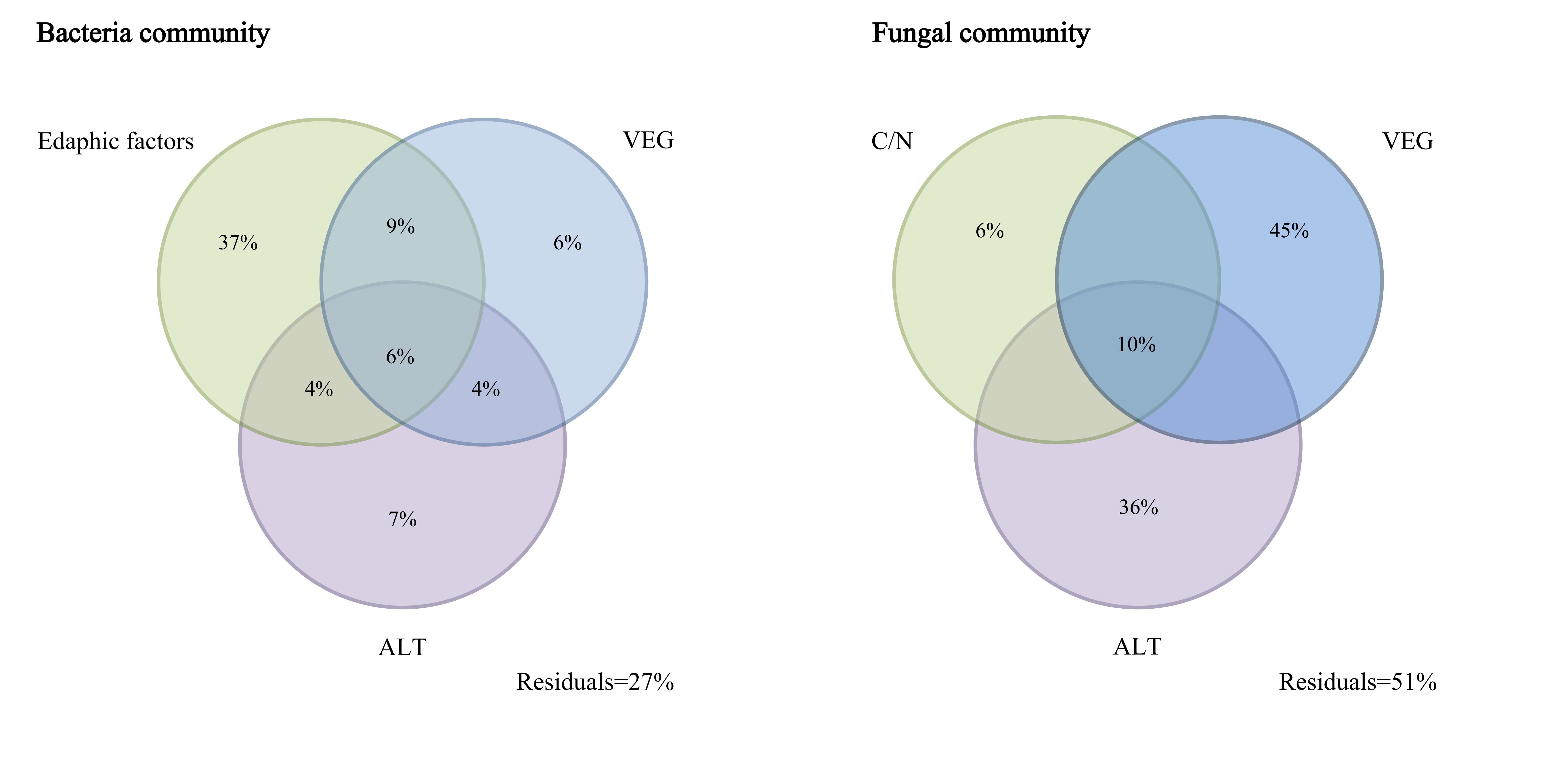

Supplement: Supplementary Figure S3 — Variance partitioning analysis (VPA) analysis of the contribution of environmental factors to bacterial communities and the contribution of environmental factors to the true community [file Image_3.jpeg]

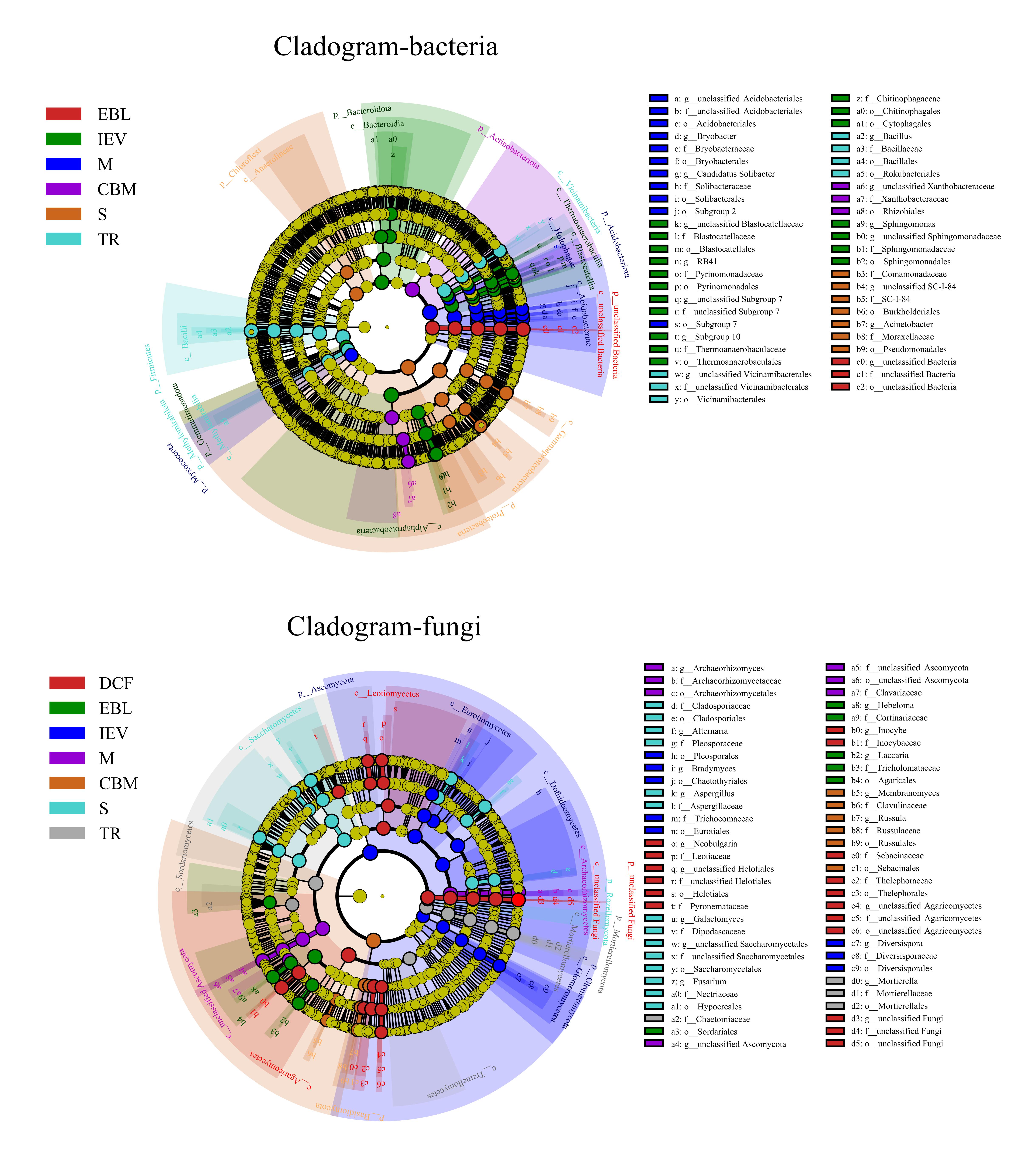

Supplement: Supplementary Figure S4 — Linear discriminant analysis effect size (LEfSe) analyses of bacterial and fungal communities associated with the vegetation type zones. TR, EBL, CBM, DCF, S, M, and IEV represent tropical rainforest, evergreen broad-leaved forest, coniferous and broad-leaved mixed forest, dark coniferous forest, shrub, meadow and ice-edge vegetation, respectively; o, f and g represent order, family and genus, respectively [file Image_4.jpeg]

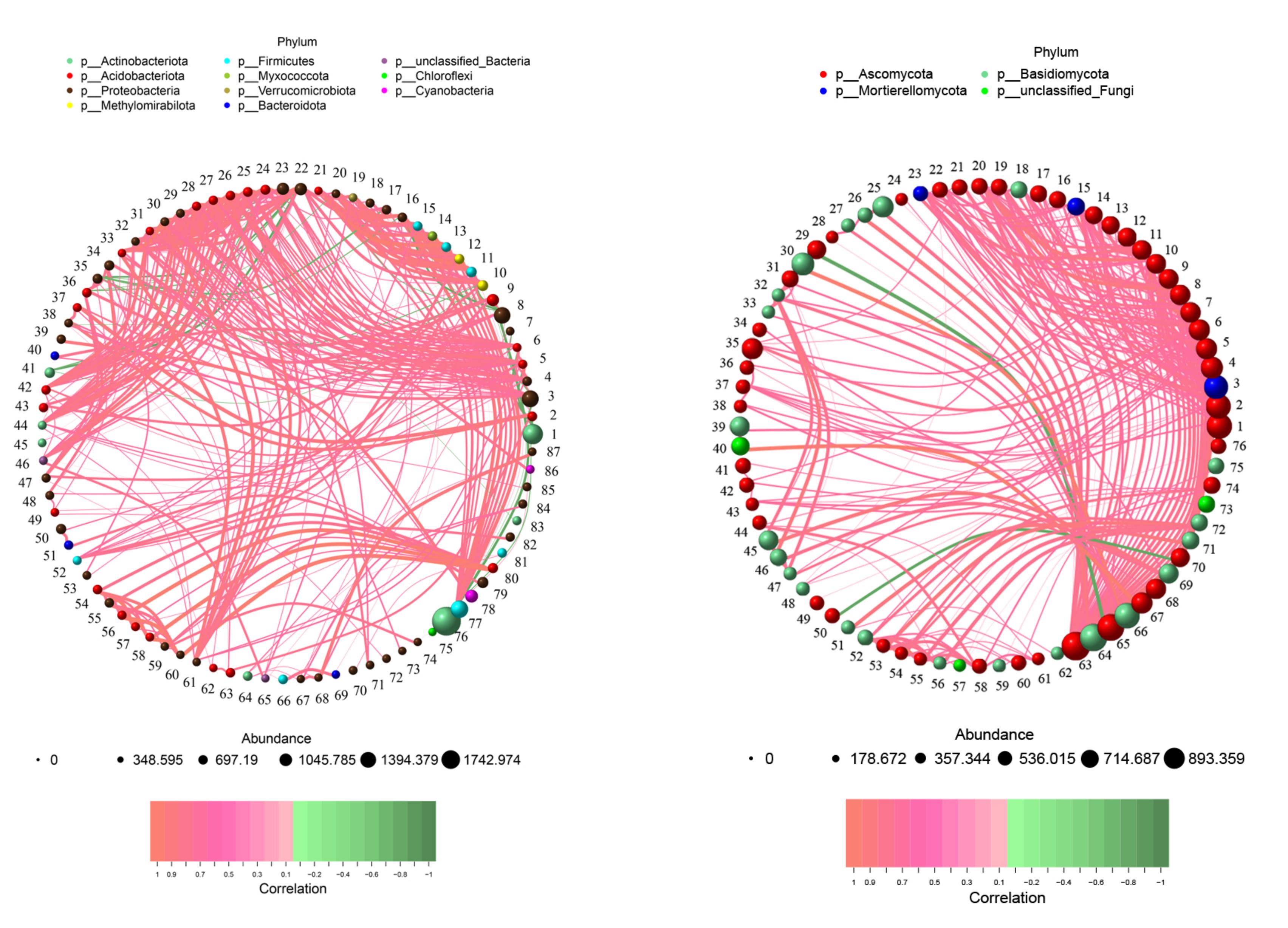

Supplement: Supplementary Figure S5 — The co-occurrence network analysis of dominant soil bacteria (A) and fungi (B) in phylum along the elevational gradient. Larger circles indicate higher relative abundance. Red lines represent positive correlation, while green lines represent negative correlation. [file Image_5.jpeg]
